# Neuromuscular electrical stimulation improves swallowing initiation in patients with post-stroke dysphagia

**DOI:** 10.3389/fnins.2022.1011824

**Published:** 2022-11-14

**Authors:** Yao-Wen Zhang, Zu-Lin Dou, Fei Zhao, Chun-Qing Xie, Jing Shi, Chen Yang, Gui-Fang Wan, Hong-Mei Wen, Pei-Rong Chen, Zhi-Ming Tang

**Affiliations:** ^1^Department of Rehabilitation Medicine, The Third Affiliated Hospital of Sun Yat-sen University, Guangzhou, China; ^2^Department of Rehabilitation Medicine, Yuedong Hospital, The Third Affiliated Hospital of Sun Yat-sen University, Meizhou, China

**Keywords:** neuromuscular electrical stimulation (NMES), delayed swallowing initiation, stroke, rehabilitation, VFSS

## Abstract

**Objective:**

More than half of post-stroke patients develop dysphagia, which manifests as delayed swallowing and is associated with a high risk of aspiration. In this study, we aimed to investigate the immediate effect of neuromuscular electrical stimulation (NMES) on swallowing initiation in post-stroke patients using videofluoroscopic swallowing study (VFSS) data.

**Materials and methods:**

This randomized, self-controlled crossover study included 35 patients with post-stroke dysphagia. All selected patients received real and sham NMES while swallowing 5 ml of thin liquid. Participants completed the conditions in random order, with a 10-min interval between conditions. The primary evaluation indicators included the Modified Barium Swallow Impairment Profile-6 (MBSImp-6) and Penetration-Aspiration Scale (PAS). Secondary indicators included oral transit time (OTT), pharyngeal transit time (PTT), and laryngeal closure duration (LCD).

**Results:**

Modified Barium Swallow Impairment Profile-6 (*P* = 0.008) and PAS (*P* < 0.001) scores were significantly lower in the Real-NMES condition than in the Sham-NMES condition. OTT (*P* < 0.001) was also significantly shorter during Real-NMES than during Sham-NMES. However, LCD (*P* = 0.225) and PTT (*P* = 0.161) did not significantly differ between the two conditions.

**Conclusion:**

Neuromuscular electrical stimulation may represent a supplementary approach for promoting early feeding training in patients with post-stroke dysphagia.

**Clinical trial registration:**

[https://clinicaltrials.gov/], identifier [ChiCTR2100052464].

## Introduction

Among the 12.42 million patients over 40 affected by stroke in China (Report on stroke prevention treatment in China Writing Group, 2020), approximately 56.9% (Zhang et al., 2020) exhibit residual swallowing dysfunction. Patients with oral and pharyngeal dysphagia mainly have difficulty initiating swallowing, which may manifest as slow oral transport, leading to difficulty in feeding by caregivers and delaying the start of pharyngeal phase, which can in turn lead to aspiration pneumonia. Delayed swallowing initiation is among the most common symptoms of swallowing disorders after stroke, with a reported incidence reaching as high as 90.1% (Yamamura et al., 2018). Delayed initiation of swallowing often results in premature spillage or pre-swallow aspiration in the early phase of swallowing, indicating that the bolus has slipped to the back of epiglottis or pyriform sinus and spilled into the laryngeal vestibule before swallowing.

Oral sensorimotor training is the most common treatment for delayed swallowing initiation (Ashford et al., 2009). However, this method is highly dependent on the patient’s level of cognitive functioning and coordination, and therapeutic outcomes vary significantly among individuals. Furthermore, the duration of traditional training methods is usually long, which means most patients will require abrosia for a few weeks before their swallowing function improves.

Neuromuscular electrical stimulation (NMES) is also a traditional treatment for dysphagia. Several studies have demonstrated the long-term therapeutic effects of NMES (2–4 weeks), which can improve swallowing function by strengthening the swallowing muscles (Alamer et al., 2020), rehabilitating the swallowing reflex, and regulating cortical excitability. Some studies have also reported that NMES can increase the sensory input of the oral pharyngeal region and enhance the sensitivity of muscle contraction (Simonelli et al., 2019; Seo et al., 2021). A study by Yamamura (2007) established a peripheral nerve stimulation system in experimental animals, including taste stimulation, temperature stimulation, and electrical stimulation (Yamamura et al., 2010). They found that they could activate swallowing by directly stimulating the peripheral nerves of the test animals. The system was verified in healthy people, and it was finally found that stimulating the healthy individual’s pharyngeal sensory area with a certain intensity of current could effectively activate its swallowing action. However, the study used a nasal invasive electrode, which can cause significant discomfort to patients, which can affect the effectiveness and safety of swallowing. In the treatment of dysphonia, NMES stimulation with front and rear opposed electrodes, as a treatment method that can produce strong sensory stimulation to the throat, has been reported by many studies (Kosztyła-Hojna et al., 2012b; Mulheren and Ludlow, 2017). Therefore, in this study, we used this modality of NMES as a pharyngeal sensory stimuli to improve swallow initiation in patients. We hypothesized that NMES have immediate effects on swallowing initiation without increasing the risk of adverse events, and we aimed to evaluate the immediate effect of NMES on swallowing function using videofluoroscopic swallowing study (VFSS) data.

## Materials and methods

### Participants

Thirty-five patients with delayed swallowing initiation after stroke were enrolled in the study. Patients were considered eligible for inclusion if they met the following criteria: All patients (age: 18–80 years) had been diagnosed with stroke (Chinese Society of Neurology, 2018). Those diagnosed with dysphagia during clinical evaluation underwent a VFSS. Delayed initiation of swallowing was identified based on Modified Barium Swallow Impairment Profile-6 (MBSImP) scores. Patients with MBSImP-6 scores below level 3 were selected for the current study. The exclusion criteria were as follows: severe diseases of the heart, liver, lung, kidney, or other vital organs; history of epilepsy after stroke; nasopharyngeal cancer or tumors of the head and neck after radiotherapy; severe listening or comprehension disorders.

The study was approved by the Medical Ethics Committee of the Third Affiliated Hospital of Sun Yat-sen University (No. [2020]02-258-01) and has been registered in the Chinese Clinical Research Registry (ChiCTR2100052464). Informed consent was obtained prior to the start of all studies.

### Study design and procedure

**FIGURE 1:**
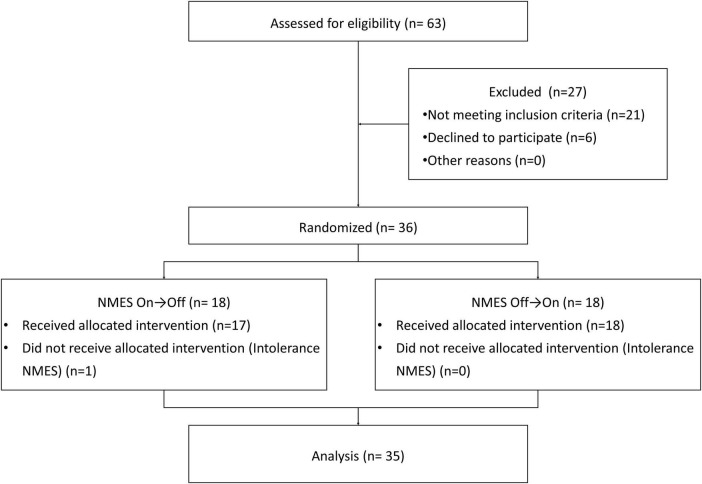
Flow chart of participant inclusion.

A randomized, self-controlled crossover design was used in this study. Each patient received both real and sham NMES (Figure 1). To eliminate order effects, 17 patients received real stimulation first, while the remaining 18 received sham stimulation first. Random numbers were generated using Microsoft Excel, and the order was secured with a person who did not participate in any of the assessments. To avoid lingering effects, the interval between the two stimulation conditions was 10 min. The participants were advised to abstain from any drugs or beverages that could impair brain function during the study.

### Neuromuscular electrical stimulation program

**FIGURE 2:**
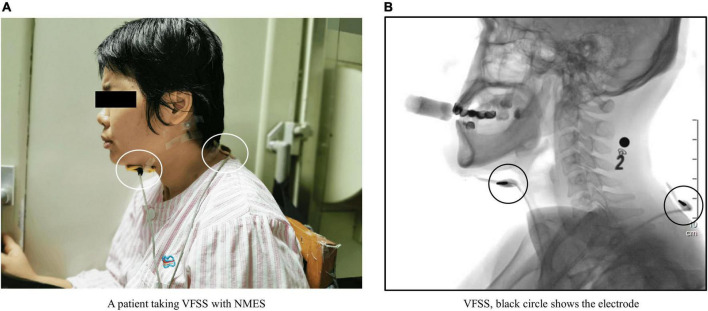
**(A)** Shows a patient undergoing videofluoroscopic swallowing studies (VFSS). The white circles indicate two electrodes placed on the patient’s neck. **(B)** Shows a VFSS image of this patient. The black circles show the electrodes observed in the VFSS image. The black number and black square at the back of the patient’s neck in panel **(B)** are markers affixed to the base of the patient’s mastoid process. The number is a steel metal number that is used to mark the food characteristics that the patient is currently eating. The black square is a steel metal ball, which is used as a plotting scale to determine the magnification of the analyzed image during quantitative analysis.

Patients were seated comfortably with the hip and knee flexed at 90 degrees, and the chair was stably fixed on the standing plane of the imaging device (Hanna and Randall, 2021), with the radiation tube located on the left side of the patient (Figure 2).

Neuromuscular electrical stimulation was delivered using a domestic YS1001P (Longest Co., Ltd, China) device, which had a continuous stimulation with a triangular amplide modulation that has a 700–1,000 ms cycle length, and a stimulation intensity of 0–30 mA. Two 5 cm × 5 cm yellow sponge electrodes were placed on the front and back of the patient’s neck, respectively. The front electrode was placed on the suprahyoid muscle group (from the lower jaw to the area above the thyroid cartilage), and the back electrode was placed near the C7 spinous process (Wang et al., 2012; Tao et al., 2014).

In the real stimulation condition, the current that would lead to obvious swallowing action was adjusted for each patient, and the average intensity was between 15 and 25 mA (Poorjavad et al., 2014). When performing Real-NMES, the device was first adjusted to a suitable current intensity, and the swallowing test was initiated 30 s after the patient had fully adapted. Real stimulation was performed during feeding with three mouthfuls of mildly thick food. The stimulation period lasted 5 min. In the sham stimulation condition, no current was applied, although other settings including the positions of the sponge electrodes and duration were the same as those used for real stimulation.

### Videofluoroscopic swallowing study procedure

Patients were asked to swallow moderately thick, mildly thick, thin, and extremely thick foods (Cichero et al., 2017) in sequence at volumes of 3, 5, 10, and 20 ml each (Rofes et al., 2012). Ten minutes after routine VFSS, all patients diagnosed with delayed swallowing initiation were asked to eat three mouthfuls of 5 ml mildly thick (honey-like, IDDSI 2) food under NMES (“on” or “off” condition), and the feeding process finished in 5 min. The order of the two feeding conditions was determined using a random number table, and the conditions were again separated by a 10-min interval. All eating processes were recorded using the VFSS system at rate of 30 frames per second.

### Evaluation of videofluoroscopic swallowing study

#### Semi-quantitative assessment

Delayed swallowing initiation was diagnosed based on MBSImP item 6 (Martin-Harris et al., 2008): Initiation of the pharyngeal swallow (IPS). For this item, scores of 0 indicate no abnormalities in swallowing initiation, which is represented by the bolus head at the region of the posterior angle of the ramus and back of the tongue at the time of the first movement of the hyoid trajectory. Scores of 1–4 indicate varying degrees of delay in initiating swallowing. A score of 1 indicates the bolus head at the valleculae at the time of the first movement of the hyoid trajectory. A score of 2 indicates that the bolus head is at the posterior laryngeal surface of the epiglottis at the first onset of hyoid excursion. A score of 3 indicates that the bolus head is in the pyriform sinus at the first hyoid excursion. A score of 4 indicates no appreciable initiation of swallowing at any bolus location.

Videofluoroscopic swallowing study data were evaluated by experienced speech therapists, who also recorded the Penetration-Aspiration Scale (PAS) score for each patient (Rosenbek et al., 1996).

#### Quantitative assessment

**FIGURE 3:**
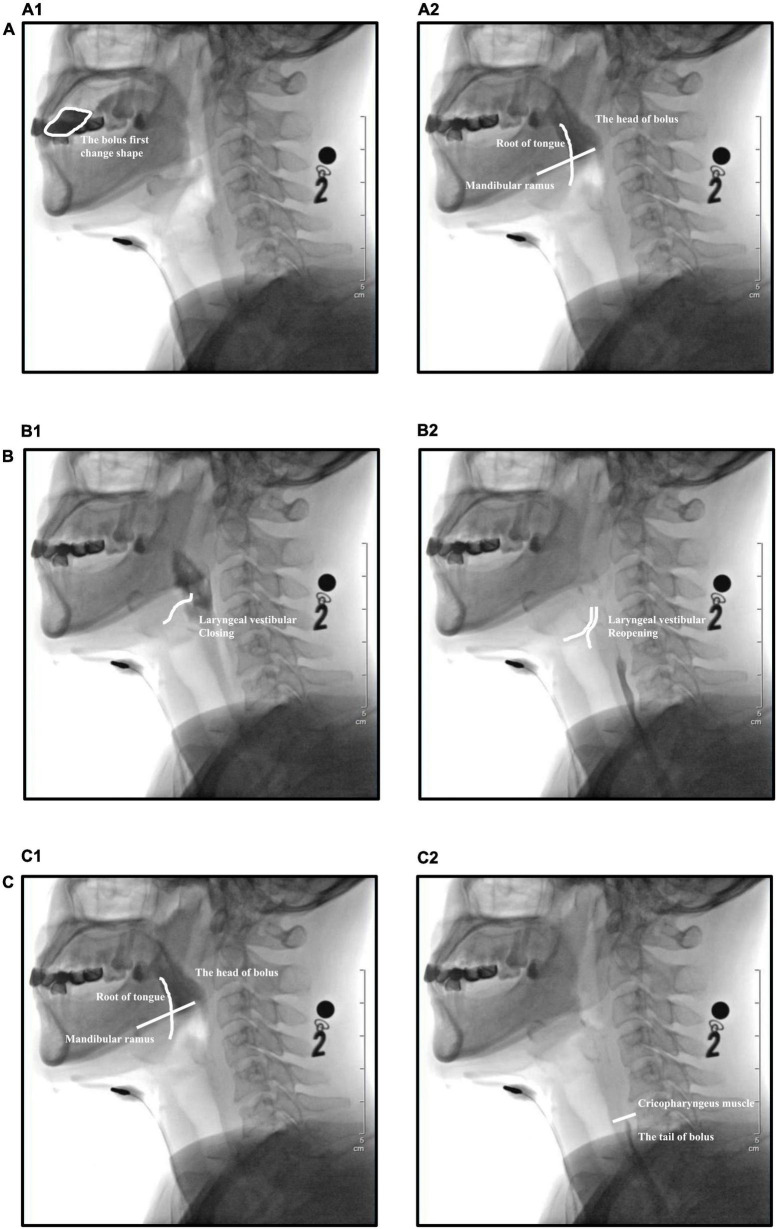
Temporal parameters evaluated during videofluoroscopic swallowing studies (VFSS). **(A)** Oral transit time (OTT), **(A1)** the bolus first changes shape when pushed by the tongue, **(A2)** the head of the bolus reaches the intersection of the mandibular ramus and the root of the tongue. **(B)** Laryngeal vestibular closure duration (LCD), **(B1)** closure of the laryngeal vestibule, **(B2)** reopening of the laryngeal vestibule. **(C)** Pharyngeal transit time (PTT), **(C1)** the head of the bolus reaches the junction of the mandibular ramus–tongue base, **(C2)** the tail of the bolus passes through the cricopharyngeal muscle.

Software (Longest, Ltd., China) was used for the quantitative analysis of VFSS data based on the following parameters (Kendall et al., 2015): oral transit time (OTT), laryngeal vestibular closure duration (LCD), and pharyngeal transit time (PTT). OTT refers to the time from the first change in bolus shape when pushed by the tongue to the time at which the head of the bolus reaches the intersection of the mandibular ramus and the root of the tongue. LCD refers to the time between the closure of the laryngeal vestibule (epiglottis inversion, laryngeal lifting, laryngeal vestibular soft tissue closure during lateral photographing) and the reopening of the laryngeal vestibule. PTT refers to the time from the head of the bolus first reaching the junction of the mandibular ramus–tongue base to the passage of the bolus tail through the cricopharyngeal muscle (Figure 3).

### Statistical analysis

We used G * Power Statistics (3.1.9.2 version) to calculate the sample size of this study according to the results of pre-experiment. To achieve 80% power and 5% statistical significance, a sample size of 35 cases was required for this study.

Statistical analyses were completed using SPSS 19.0. Data normality and homogeneity of variance were examined using the Shapiro–Wilk test. Non-parametric data were described as medians (IQR: 25,75%), while parametric data were described as the mean ± SD. Wilcoxon signed-rank tests were performed to compare IPS and PAS. A Holm–Bonferroni adjustment was used to correct for multiple comparisons and adjust the familywise error rate. Pearson correlation coefficients were used to determine effect size. Temporal parameters (OTT, LCD, and PTT) were compared using paired *t*-tests. The level of statistical significance was set at *p* < 0.05, and adjusted *p*-values were reported.

## Results

### Participant demographics

In the clinical evaluation process (Figure 1), 63 patients were identified, and 36 of these patients met our inclusion criteria. Except for one patient who dropped out of the study due to intolerance to NMES, the other 35 participants (23 men, 12 women) meeting the inclusion criteria completed all experimental procedures. Participant demographics, including information regarding stroke subtype, disease duration, and stroke location, are included in Table 1. The mean age among included participants was 56 ± 12.31 years, and the average disease duration was 67 ± 23.77 days. Stroke subtypes included cerebral hemorrhage (*n* = 20, 57.1%) and cerebral infarction (*n* = 15, 42.9%). The majority of participants had experienced pure cortical stroke (*n* = 13, 37.1%) or brainstem stroke (*n* = 19, 54.3%), and only a few participants had strokes in two locations (*n* = 3, 8.6%).

**TABLE 1:** Participant characteristics.

Age (years)		56 ± 12.31
Sex	Male	23 (65.7%)
	Female	12 (34.3%)
Disease duration (days)		67 ± 23.77
Stroke subtype	Hemorrhage	20 (57.1%)
	Infarction	15 (42.9%)
Stroke location	Cortex	13 (37.1%)
	Brainstem	19 (54.3%)
	Both	3 (8.6%)

### Semi-quantitative assessment results

**FIGURE 4:**
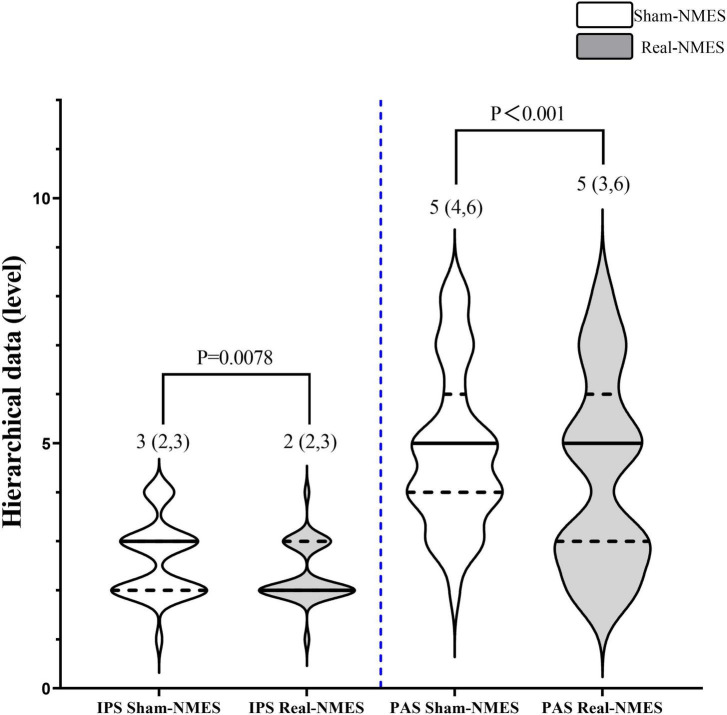
Comparisons of hierarchical data between Sham-NMES and Real-NMES. IPS, initiation of the pharyngeal swallow; PAS, Penetration–Aspiration Scale; NMES, neuromuscular electrical stimulation.

Changes in hierarchical data are shown in Figure 4. After NMES, eight patients (23%) exhibited improvements in the IPS rating, 2(2,2.75) in Real-NMES and 3.5(3,4) in Shame-NMES, while 17 patients (49%) exhibited improvements in the PAS rating, 3(2,3) in Real-NMES and 4(3,4) in Shame-NMES. On average, the IPS score for Real-NMES was 2 (IQR: 2,3), which was significantly lower than that for Sham-NMES [3 (IQR: 2,3); *Z* = −2.64, *P* = 0.008, Figure 4)]. Meanwhile, the PAS score was significantly lower for the Real-NMES condition [5 (IQR: 3,6)] than for the Sham-NMES condition [5 (IQR: 4,6); Z = −4.123, *P* < 0.001, Figure 4].

### Quantitative assessment results

**FIGURE 5:**
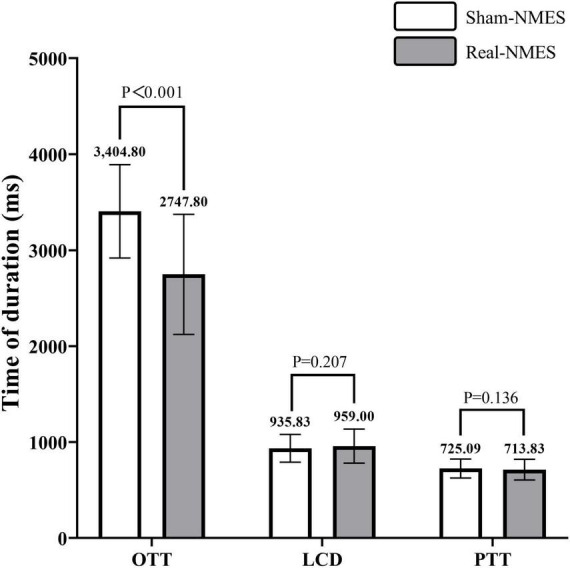
Comparisons of temporal parameters between Sham-NMES and Real-NMES. OTT, oral transit time; LCD, laryngeal vestibular closure duration; PTT, Pharyngeal transit time, NMES, neuromuscular electrical stimulation.

Oral transit time was significantly shorted in the Real-NMES condition (2,747.80 ± 625.73 ms) than in the Sham-NMES condition (3,404.80 ± 486.71 ms; *t* = 19.031, *P* < 0.001, Figure 5). There was no significant difference in LCD between Sham-NMES (935.83 ± 144.63 ms) and Real-NMES (959.00 ± 177.32 ms; *t* = −1.285, *P* = 0.207). There was also no significant difference in PTT between Sham-NMES (725.09 ± 98.21 ms) and Real-NMES (713.83 ± 107.68 ms; *t* = 1.526, *P* = 0.136, Figure 5).

## Discussion

In this study, we performed quantitative analyses of VFSS data to assess swallowing performance in patients with delayed swallowing initiation after stroke when stimulation with NMES was turned “on” or “off.” Our findings indicated that use of NMES significantly shortened OTT and improved IPS while decreasing the risk of penetration and aspiration. These results suggest that NMES exerts an immediate positive effect on the initiation of swallowing in patients with dysphagia after stroke.

Approximately 70% of patients with delayed swallowing initiation after stroke exhibit the “premature spillage” (Warnecke et al., 2021) phenomenon, in which the ready-to-eat bolus has slipped into the posterior wall of the epiglottis or the area of the piriform sinus before swallowing. This allows the bolus to overflow into the vestibule of the larynx, causing penetration and aspiration. During the eating process, foods with lower viscosity are more likely to cause penetration and aspiration (Newman et al., 2016). As a result, caregivers often thicken the food of the dysphagia patients (Watanabe et al., 2018), but the thickened food can make it more difficult for the patient to transport it through the mouth (Jeong et al., 2021), which can cause the food to stay in their mouth longer. For patients with oral transit difficulties and delayed swallowing initiation, the longer the food stays in the oral cavity, the higher the degree of hydrolysis by salivary amylase even if the food is thickened (Abu-Hasan et al., 2014; Sole et al., 2020). Therefore, shortening the OTT can prevent food from being hydrolyzed and thinned by saliva in the oral cavity, thereby maintaining the food’s properties and consistency and reducing the risk of penetration and aspiration during swallowing.

Our findings demonstrated that NMES can improve the patient’s ability to initiate swallowing in the pharyngeal phase and reduce the risk of penetration and aspiration in the eating process, suggesting that it can aid in early feeding training for patients with dysphagia following stroke. In clinical settings, oral sensory stimulation combined with electrical stimulation is the most method applied in patients with dysphagia patients after stroke (Guillen-Sola et al., 2017; Wakabayashi et al., 2018), especially those with cognitive impairment. However, due to limitations in cognitive function and cooperation ability, the treatment cycle is usually long (Park et al., 2019), therefore, these patients need to stop oral feeding for a considerable period of time, which often leads to disuse atrophy of the swallowing muscles (Duranceau et al., 1983), which leads to further decline in swallowing function. Previous studies have mainly focused on the long-term effects of NMES treatment. The general treatment time is about 4–6 weeks, and the observation results are not the same (Speyer et al., 2022). The results of Jo Frost’s research (Frost et al., 2018) show that NMES combined with traditional oral sensorimotor training has a positive effect on the recovery of patients’ swallowing function, which can improve patients’ oral feeding function and reduce the risk of leakage and aspiration. To the contrary, Carnaby et al. (2020) reports that McNeill Dysphagia Therapy (MDPT) alone was more effective in reducing in dysphagia severity, improved oral intake and earlier return to pre-stroke diet in patients with dysphagia than MDPT combined with NMES, they believe that NMES has a detrimental effect on some patients with dysphagia. Combined with the disuse atrophy mentioned above, the patient’s swallowing function will continue to decline, so it is not difficult to understand the difference in the therapeutic effect of this long-term NMES stimulation. This study mainly focused on the immediate effect of this new NMES stimulation method, which can improve the patient’s swallow initiation function and reduce the probability of aspiration, so we consider it as a substitute for therapeutic eating training. Compensation means to help patients carry out feeding training as early as possible, so as to maintain and improve the patient’s existing swallowing function as much as possible.

In this study, NMES did not induce significant changes in LCD or PTT. Huang et al. (2014) reported that LCD was significantly shorter in patients with stroke than in healthy controls, indicating that the time for the laryngeal vestibule to close during the swallowing process is insufficient in patients with stroke, which increases the risk of penetration and aspiration. However, studies have noted that improving the initiation of pharyngeal swallowing can effectively prevent aspiration induced by insufficient LCD, consistent with the current results (Yang et al., 2015). Although LCD did not change significantly during NMES stimulation, our patients experienced improvements in PAS scores. Park et al. (2010) reported that supraglottic swallowing can significantly prolong the LCD in patients with dysphagia, thereby reducing the risk of aspiration, suggesting that improvements in LCD can be used as a therapeutic indicator during feeding training. Moreover, if NMES cannot extend the LCD immediately, feeding training combined with supractic swallowing may more effectively reduce the risk of penetration and aspiration, although this must be explored in future studies.

In this study, we also observed no significant, immediate changes in PTT with NMES. This result differs from those reported in previous studies (Park et al., 2019), which have indicated that long-term NMES treatment can effectively improve PTT. Verin et al. (2011) demonstrated that the long-term effect of NMES on PTT is related to the functional reorganization of the pharyngeal motor cortex. Long-term sensory stimulation can activate the neuroplasticity of the swallowing-related cortex in patients with stroke; however, our findings suggest that such changes are unlikely to occur in the immediate period following NMES.

The anteroposterior NMES electrical stimulation device used in this study is most commonly used in the treatment of some vocal cord paralysis (Maryn et al., 2003) or voice disorders (Kosztyła-Hojna et al., 2012a). Its therapeutic effect is mainly to generate a current path through two electrodes facing each other, which can stimulate the deep muscles and nerves of the patient’s pharynx, including the recurrent laryngeal nerve and the pharyngeal nerve. Therefore, the mechanism of NMES’s effect on OTT and IPS of patients in this study may also be here. NMES device enhances the patient’s pharyngeal sensory function by stimulating the patient’s recurrent laryngeal nerve and pharyngeal nerve, thereby improving the patient’s feeding performance. However, this is only a conjecture based on previous studies, and further research on the mechanism of action of this NMES stimulation is needed in the future to better explain its effect on the swallowing initiation function of patients.

This study had some limitations. First, the optimal electrical stimulation site and stimulation parameters were not discussed in depth. At present, the current intensity is generally adjusted to the maximum tolerance of the patient or the intensity that produces obvious swallowing actions, highlighting the need for further studies of optimal parameters. Second, we did not analyze the effect of NMES combined with food boluses of different viscosity and volume on immediate changes in the patient’s eating function. Lastly, we did not investigate the advantages of NMES combined with eating training when compared with pure NMES. In the future, more in-depth research is required to elucidate the long-term effects of NMES combined with eating training.

## Conclusion

The present results suggest that NMES can improve oral transit and swallowing initiation while decreasing the risk of penetration and aspiration in patients with post-stroke dysphagia. Thus, NMES may aid in promoting early therapeutic feeding following stroke.

## Data availability statement

The original contributions presented in this study are included in the article/Supplementary material, further inquiries can be directed to the corresponding author.

## Ethics statement

The studies involving human participants were reviewed and approved by the Ethics Committee of The Third Affiliated Hospital of Sun Yat-sen University. The patients/participants provided their written informed consent to participate in this study.

## Author contributions

Z-MT and Z-LD: conceptualization. Y-WZ, G-FW, and H-MW: methodology. Y-WZ and C-QX: assessment. C-QX and P-RC: therapy in charge. Y-WZ and Z-MT: formal analysis and investigation and writing—review and editing. Y-WZ and FZ: writing—original draft preparation. Z-LD: funding acquisition. Z-MT: supervision. All authors contributed to the study conception and design.
