# Supplementary material for: Neuromuscular electrical stimulation improves swallowing initiation in patients with post-stroke dysphagia
Source: Front Neurosci. 2022 Nov 14;16:1011824. doi: 10.3389/fnins.2022.1011824 (PMC9704362; doi:10.3389/fnins.2022.1011824)
Supplement: Supplementary file 1 [file Table_1.DOCX]

**Supplement table 1. VFSS descriptive statistics of Sham-NMES and Real-NMES**

|  | IPS | PAS | OTT(ms) | LCD(ms) | PTT(ms) |
| --- | --- | --- | --- | --- | --- |
| Sham-NMES | 3 (2, 3) | 5 (4, 6) | 3404.80±486.71 | 935.83±144.63 | 725.09±98.21 |
| Realt-NMES | 2 (2, 3) | 5 (3, 6) | 2747.80±625.73 | 959.00±177.32 | 713.83±107.68 |
| Z / t | -2.640 | -4.123 | 19.031 | -1.285 | 1.526 |
| P | 0.008 | ＜0.001 | ＜0.001 | 0.207 | 0.136 |
| IPS: Initiation of the pharyngeal swallow; PAS: Penetration-Aspiration Scale(PAS); OTT: Oral Transit Time; PTT: Pharyngeal Transit Time; LCD: Laryngeal Closure Duration | | | | | |
